# Low-temperature exposure has immediate and lasting effects on the stress tolerance, chemotaxis and proteome of entomopathogenic nematodes

**DOI:** 10.1017/S0031182022001445

**Published:** 2023-01

**Authors:** Peter E. Lillis, Ian P. Kennedy, James C. Carolan, Christine T. Griffin

**Affiliations:** Department of Biology, Maynooth University, Maynooth, County Kildare, Ireland

**Keywords:** Behaviour, biocontrol, dauer, *Heterorhabditis megidis*, infective juvenile, infective larva, *Steinernema carpocapsae*, storage, survival

## Abstract

Temperature is one of the most important factors affecting soil organisms, including the infective stages of parasites and entomopathogenic nematodes, which are important biological control agents. We investigated the response of 2 species of entomopathogenic nematodes to different storage regimes: cold (9°C), culture temperature (20°C) and temperature swapped from 9 to 20°C. For *Steinernema carpocapsae*, cold storage had profound effects on chemotaxis, stress tolerance and protein expression that were retained in temperature-swapped individuals. These effects included reversal of chemotactic response for 3 (prenol, methyl salicylate and hexanol) of the 4 chemicals tested, and enhanced tolerance to freezing (−10°C) and desiccation (75% RH). Label-free quantitative proteomics showed that cold storage induced widespread changes in *S. carpocapsae*, including an increase in heat-shock proteins and late embryogenesis abundant proteins. For *Heterorhabditis megidis*, cold storage had a less dramatic effect on chemotaxis (as previously shown for proteomic expression) and changes were not maintained on return to 20°C. Thus, cold temperature exposure has significant effects on entomopathogenic nematodes, but the nature of the change depends on the species. *Steinernema carpocapsae*, in particular, displays significant plasticity, and its behaviour and stress tolerance may be manipulated by brief exposure to low temperatures, with implications for its use as a biological control agent.

## Graphical abstract

**Figure:**
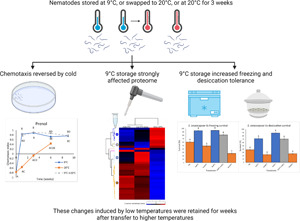

## Introduction

Many nematode parasites of plants and animals achieve transmission by means of a free-living infective larva or infective juvenile (IJ). Temperature can profoundly impact the success of IJs in many ways, with moderate temperatures affecting the rates of dispersal or host finding, while extreme temperatures are lethal or damaging (Grewal *et al*., 1994; O'Connor *et al*., 2006; Bryant and Hallem, 2018; Aleuy and Kutz, 2020). Less extreme temperatures may also affect survival, for example, by slowing metabolism (Andaló *et al*., 2011). Conditions experienced in the past may affect current activities; for species occurring at higher latitudes, the most widely documented effects are those of low temperature which may protect against subsequent more damaging conditions through acclimatization, or act to induce diapause or quiescence (Sommerville and Davey, 2002; McSorley, 2003; Zhao *et al*., 2007; Evans and Perry, 2009; Aleuy and Kutz, 2020). Here, we explore the effects of prior temperature conditions on entomopathogenic nematodes (EPN).

EPN *Steinernema* and *Heterorhabditis* have received much attention in recent years due to their usefulness both as model parasites and as biological control agents (Hallem *et al*., 2007; Koppenhöfer *et al*., 2020). Their IJs seek out and infect living insects, typically in soil. The IJ of EPN is similar to that of other plant and animal parasites (where present), and to the dauer juvenile of free-living nematodes such as *C*aenorhabditis *elegans* (Bubrig and Fierst, 2021), being a developmentally arrested, non-feeding stage with sealed mouth and anus, depending on lipid and glycogen stores for survival. The IJ, being a dauer stage, is more resistant than other life stages to abiotic stressors such as ultraviolet radiation, desiccation and extreme temperatures (Gaugler *et al*., 1992; Hibshman *et al*., 2020). EPN have a mutualistic relationship with insect pathogenic bacteria (*Xenorhabdus* spp. for steinernematids and *Photorhabdus* spp. for heterorhabditids), which are released by the IJ once inside a host (Forst *et al*., 1997). The bacteria multiply, killing the insect (in conjunction with the nematodes) and converting it to a food source for 1 or more generations of nematodes; newly developed IJs emerge from depleted cadavers and disperse in search of new hosts. *Heterorhabditis* and *Steinernema* are not closely related (Blaxter *et al*., 1998), and have convergently evolved the entomopathogenic lifestyle (Poinar, 1993). EPN occur widely in nature, and several species are promising biocontrol agents, which are mass-produced and then stored until applied against insect pests (Lacey *et al*., 2001).

The effects of storage temperature and time (‘age’) on behaviours such as dispersal and infection have been documented for several EPN species (Fan and Hominick, 1991; Griffin, 1996; Yoder *et al*., 2004; Koppenhöfer *et al*., 2013). More recently it has been shown that culture and storage temperatures have profound effects on the chemotaxis of both EPN and animal parasites, including reversals of valence between attraction and repulsion to the same chemical (Lee *et al*., 2016), which could impact host-finding and foraging behaviour. Prior exposure to low temperatures can also enhance IJs' resistance to freezing (Brown and Gaugler, 1996; Ali and Wharton, 2013) and desiccation (Jagdale and Grewal, 2007).

This study aims to investigate the effects of temperature and time on the behaviour, stress tolerance and proteome of *Steinernema carpocapsae*, the best studied and most economically important EPN species. We also include *Heterorhabditis megidis* in the behavioural assays; the behaviour of this species has been extensively studied with respect to storage temperature and time (Griffin, 1996; O'Leary *et al*., 1998; Fitters and Griffin, 2004; Guy *et al*., 2017). We have recently shown, using quantitative label-free proteomics, that the proteomes of *S. carpocapsae* and *H. megidis* IJs differ in their response to temperature: when newly emerged IJs were transferred from a culture temperature of 20 to 9°C, the proteome of *S. carpocapsae* underwent profound changes that were maintained during further storage at that temperature, while the proteome of *H. megidis* underwent more gradual changes over time at both 20 and 9°C (Lillis *et al*., 2022). Here, we extend our exploration of the effects of storage temperature and time on these 2 species, using similar conditions to those of Lillis *et al*. (2022) but including phenotypic assays (chemotaxis and, for *S. carpocapsae*, stress tolerance), and investigating whether cold-induced changes are retained when IJs are returned to their culture temperature. Specific objectives were (1) following Lee *et al*. (2016), to explore how olfactory responses of both species change over time at different storage temperatures; (2) to test how 9°C storage protects *S. carpocapsae* IJs against stress (freezing and desiccation); (3) to investigate whether changes in olfaction and stress tolerance induced in *S. carpocapsae* by short-term storage at 9°C are maintained following return to culture temperature; and (4) to compare the proteome of such temperature-swapped IJs with that of IJs maintained in constant conditions.

## Materials and methods

### Nematode culturing and conditioning

*Heterorhabditis megidis* UK211 and *S. carpocapsae* All (both of which had been maintained in laboratory culture for several years) were cultured in last instar *Galleria mellonella* larvae (Mealworm Company, Sheffield, UK) using methods outlined in Woodring and Kaya (1988), at 20°C, with an inoculum density of 100 IJs per insect. The larvae died after 2–3 days, and cadavers were placed on White traps (White, 1927) and monitored daily. After the first emergence of IJs, the White trap water was replaced with fresh sterile tap water. IJs that emerged into the water over 3–4 days were collected and rinsed 3 times by sedimentation. The IJs were stored at 1000 IJs mL^−1^ in sterile tap water in 35 mL aliquots in lidded plastic tubs (9 cm diameter). IJs were placed at constant temperatures and assayed at intervals. In some experiments, IJs were transferred from one temperature to another. Each tub of IJs was used once only.

### Chemotaxis assays

Chemotaxis assays (Bargmann *et al*., 1993) were conducted on 60 mm Petri dishes with 20 mL of 2% nutrient agar. Test stimulus (5 *μ*L) was added to a circle (1 cm diameter) on one side of the plate, and 5 *μ*L of the appropriate diluent was added to a control circle on the opposite side. Sodium azide (2 *μ*L) was added to each circle, to anaesthetize nematodes arriving there (Bargmann *et al*., 1993). IJs were concentrated by sedimentation and approximately 100–250 IJs in 2 *μ*L were added to the centre of the plate. IJs were allowed to migrate for 1 h at 20°C. The chemotaxis index (CI) was calculated as (number of IJs in treatment circle–number of IJs in control circle)/total number of IJs in both circles. Plates which had fewer than 5 IJs in either scoring region were discounted to prevent small numbers from skewing the data (Hallem *et al*., 2011).

Putative attractants and repellents were chosen based on the literature: methyl salicylate (Chaisson and Hallem, 2012), acetone (O'Halloran and Burnell, 2003), prenol (Baiocchi *et al*., 2017, 2019; Kin *et al*., 2019) and hexanol (O'Halloran and Burnell, 2003; Chaisson and Hallem, 2012). Prenol (3-methyl-2-buten-1-ol) was diluted to 2 m, by mixing 203 *μ*L of 99.9% prenol (Sigma-Aldrich, UK) with 797 *μ*L of ethanol as per Baiocchi *et al*. (2017). Dilutions (1 in 10) of acetone, hexanol and methyl salicylate were made in paraffin oil. Sodium azide (1 m) was prepared by transferring crystalline sodium azide to 1 mL MilliQ water.

There were 3 experiments utilizing chemotaxis assays. In experiment 1, IJs of *S. carpocapsae* and *H. megidis* were assayed at time 0 (immediately after collection and washing), and after 1, 3, 6 and 9 weeks at 9°C, and 1, 3 and 6 weeks at 20°C. An additional treatment was included for *S. carpocapsae*: after IJs were stored at 9°C for 1 week, they were transferred to 20°C, and assayed after 1 day, and 2, 5 and 8 weeks (corresponding to a total time of 3, 6 or 9 weeks from time 0).

In experiment 2, odours which elicited significantly different CIs at the 3-week timepoint in experiment 1 were chosen to assay the effect of brief exposure to low temperatures on the CI, both immediately and after subsequent storage at 20°C. Methyl salicylate and prenol were used for *H. megidis* and hexanol and prenol were used for *S. carpocapsae*. IJs were assayed at time 0 and after 1 day, 1 week or 3 weeks at 9°C, or 3 weeks at 20°C. In addition, after 1 day or 1 week at 9°C, IJs were transferred to 20°C and assayed 3 weeks from the start of the experiment. *Steinernema carpocapsae* IJs were also assayed and transferred to 20°C after 3 h, due to the plasticity of their chemotactic responses.

In experiment 3, IJs were assayed at time 0, and after 1 and 3 weeks at 9, 12, 15 and 20°C. As above-mentioned, chemotaxis was assessed against odours which give strong responses, methyl salicylate and prenol for *H. megidis*, and hexanol and prenol for *S. carpocapsae* IJs.

In each experiment, there were 3 culture batches of each species with either 5 (experiment 1) or 10 (experiments 2 and 3) assay plates per batch to give a total of 15 or 30 plates per treatment at each timepoint, respectively. An exception was for IJs transferred from 9 to 20°C in experiment 1, where there was a total of 5 plates per timepoint. Experiment 2 was repeated with 3 culture batches and 10 plates per batch in each run.

### Freezing and desiccation assays

Assays were adapted from O'Leary *et al*. (1998) and Adhikari *et al*. (2010). Sterile tap water (50 *μ*L) containing approximately 50 IJs was pipetted onto Whatman filter paper (1 cm diameter) in a 3 cm Petri dish. Plates (without lids) were transferred to either −10°C for 6 h or 75% RH for 5 days. These conditions were chosen to give approximately 50% survival in freshly harvested (time 0) IJs. Desiccation chambers were maintained at 75% RH using supersaturated sodium chloride solutions (Winston and Bates, 1960). After the assay time, 20 mL of sterile tap water was added and IJ survival was assessed after 24 h. Experiments were conducted twice, with 3 culture batches of nematodes in each repeat of the experiment. Five plates were tested per batch of nematodes, to give a total of 30 plates per storage treatment.

### Statistical analysis

Statistics were carried out in Graphpad Prism v9.0.1. Kruskal–Wallis tests were performed on data at the significance level of *P* < 0.05, with Dunn's multiple comparison *post hoc* tests to identify groups which were significantly different.

### Protein sample preparation

The contents of a tub of IJs were sedimented in a 50 mL Falcon tube at their storage temperature. The pelleted IJs (150 *μ*L) were transferred to a 1.5 mL Eppendorf tube and snap frozen in liquid nitrogen. Each sample was homogenized in lysis buffer, containing 6 m urea, 2 m thiourea and a protease inhibitor cocktail (cOmplete, Mini Protease Inhibitor Cocktail, Merck), centrifuged at 10 000 × ***g*** for 1 min, and snap frozen. This step was repeated 3 times to ensure complete homogenization. Protein content was then quantified using Qubit (Invitrogen), following the manufacturer's instructions. Protein (100 *μ*g) was purified using a 2D Clean Up Kit (GE Healthcare) according to the manufacturer's instructions. The resulting pellets were stored in the kit's wash solution at −20°C until the last samples were collected, then all were centrifuged at 13 000 × ***g*** for 5 min and the resulting pellets were resuspended in 50 *μ*L of resuspension buffer (6 m urea, 2 m thiourea, 0.1 m Tris-HCl, pH 8). A volume of 20 *μ*L was aliquoted from each sample for reduction, alkylation and digestion. One hundred and five microlitres of ammonium bicarbonate (50 mm) and 1 *μ*L of dithiothreitol were added and the samples were incubated at 56°C for 20 min. Once cooled, the samples were alkylated with 2.7 *μ*L of iodoacetamide in the dark.

One microlitre of 1% (w/v) solution of ProteaseMax (Promega) and 0.5 *μ*g *μ*L^−1^ trypsin (Promega) were added to the samples and incubated at 37°C for a minimum of 16 h. Samples were removed from 37°C, centrifuged briefly and acidified with 1 *μ*L of trifluoroacetic acid (TFA) for 5 min at room temperature (20–25°C). Samples were centrifuged at 13 000 × ***g*** for 10 min and the supernatant was purified using C18 Spin Columns (Pierce, Thermo Fisher Scientific) following the manufacturer's instructions and then lyophilized in a Speedyvac concentrator (Thermo Scientific Savant DNA120). Samples were resuspended in a loading buffer (2% v/v acetonitrile, 0.05% v/v TFA) and 1 *μ*g per sample was loaded on a QExactive (Thermo Fisher Scientific) high-resolution accurate mass spectrometer connected to a Dionex Ultimate 3000 (RSLCnano) chromatography system. Peptides were separated over a 2–40% gradient of acetonitrile on a Thermo Fisher EASY-Spray, PepMap RSLC C18 column (500 mm length, 75 mm ID), using a reverse-phase gradient at a flow rate of 250 nL min^−1^ over 125 min. All data were acquired over 105 min with the mass spectrometer operating in automatic data-dependent switching mode. A full mass spectrometry (MS) scan at 140 000 resolution and a range of 300–1700 *m*/*z* was followed by an MS/MS scan, resolution 17 500 and a range of 200–2000 *m*/*z*, selecting the 15 most intense ions prior to MS/MS. There were 4 biological replicates (tubs of IJs) per storage treatment.

### Proteomic data processing

Protein identification and label-free quantification (LFQ) normalization of MS/MS data were performed using Max-Quant v1.6.3.3 (http://www.maxquant.org) following the general procedures and settings outlined in Hubner *et al*. (2010). The Andromeda search algorithm (Cox *et al*., 2011) incorporated in the MaxQuant software was used to correlate MS/MS data for *S. carpocapsae* against the predicted protein datasets derived from the *S. carpocapsae* genome (Serra *et al*., 2019).

Normalized LFQ intensities were used to quantify protein abundances, and the data were filtered to remove contaminants. The LFQ intensities were log_2_ transformed, and each replicate was renamed to their respective groups (e.g. 3wks9°C for proteins from IJs stored at 9°C for 3 weeks). Only proteins found in 3 replicates of at least 1 group were retained. A data imputation step replaced missing values with the values of low abundant proteins chosen randomly from a distribution specified by a downshift of 2 times the mean standard deviation (s.d.) and a width of 0.3 times the s.d.

A principal component analysis (PCA) was initially performed on the normalized intensity values of all replicates. However, a number of outliers were identified, resulting in 3 replicates in each treatment group in the final datasets for analysis.

An analysis of variance (ANOVA) was performed on all groups using a Benjamini–Hochberg false discovery rate of <1% to select proteins for *Z*-score normalization. These ANOVA significant proteins were used for hierarchical clustering of samples using Euclidean distance and average linkage pre-processed with *K* means.

Volcano plots were generated in Perseus by plotting negative log *P* values on the *y*-axis and log_2_-fold transformed differences on the *x*-axis for each comparison. Pairwise *t*-tests were performed comparing each of the cold-stored treatments (IJs stored at 9°C for 1 week and transferred to 20°C for 2 weeks, and IJs stored at 9°C for 3 weeks) to the IJs maintained at 20°C for 3 weeks, to visualize the effect of temperature change on the IJ proteome. Statistically significant (SS; *P* < 0.05) and differentially abundant (DA; fold change of 1.5) proteins were identified as SSDAs and selected for further analysis.

The genome of *S. carpocapsae* has been recently sequenced (Serra *et al*., 2019), and the protein file was downloaded from Wormbase Parasite (https://parasite.wormbase.org/Steinernema_carpocapsae_prjna202318/Info/Index) and used for detection of peptides.

The MS proteomics data and MaxQuant search output files have been deposited to the ProteomeXchange Consortium (Côté *et al*., 2012) *via* the PRIDE partner repository with the dataset identifier PXD027609.

## Results

### Effect of storage at constant temperatures (9 and 20°C) on chemotaxis

**Fig. 1.**
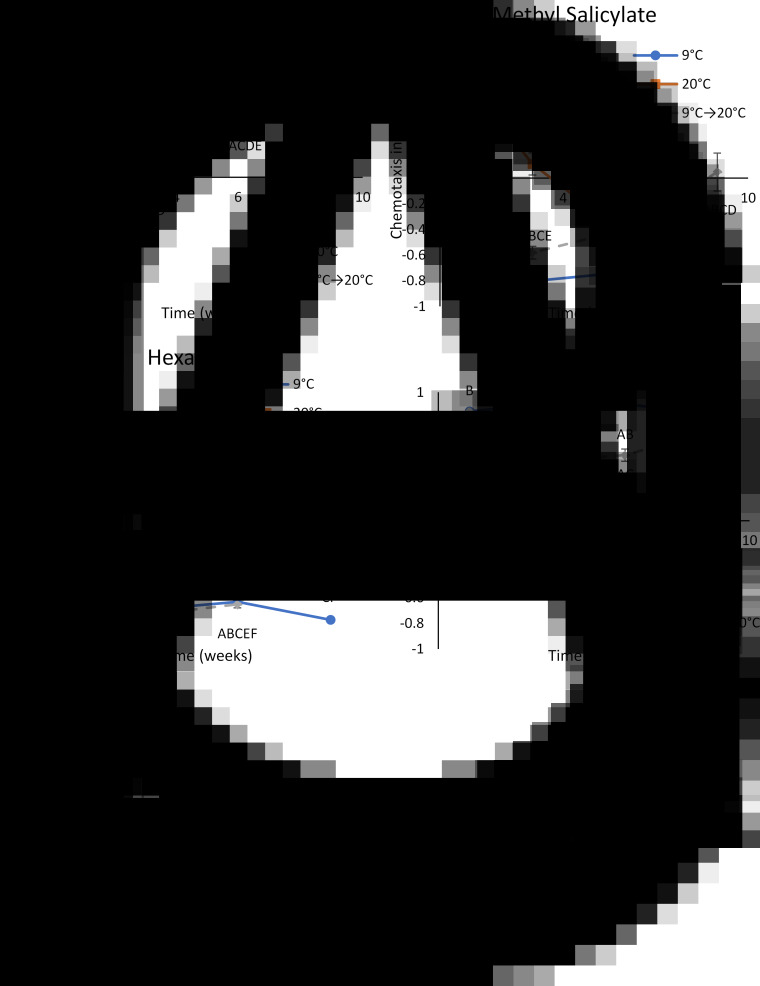
Chemotaxis index (mean ± s.e.) of *S. carpocapsae* IJs stored at 9 and 20°C, and those placed at 9°C for 1 week and transferred to 20°C in response to 4 odorants. Within a panel, values accompanied by the same letter are not significantly different (*P* < 0.05, Dunn's multiple comparisons).

**Fig. 2.**
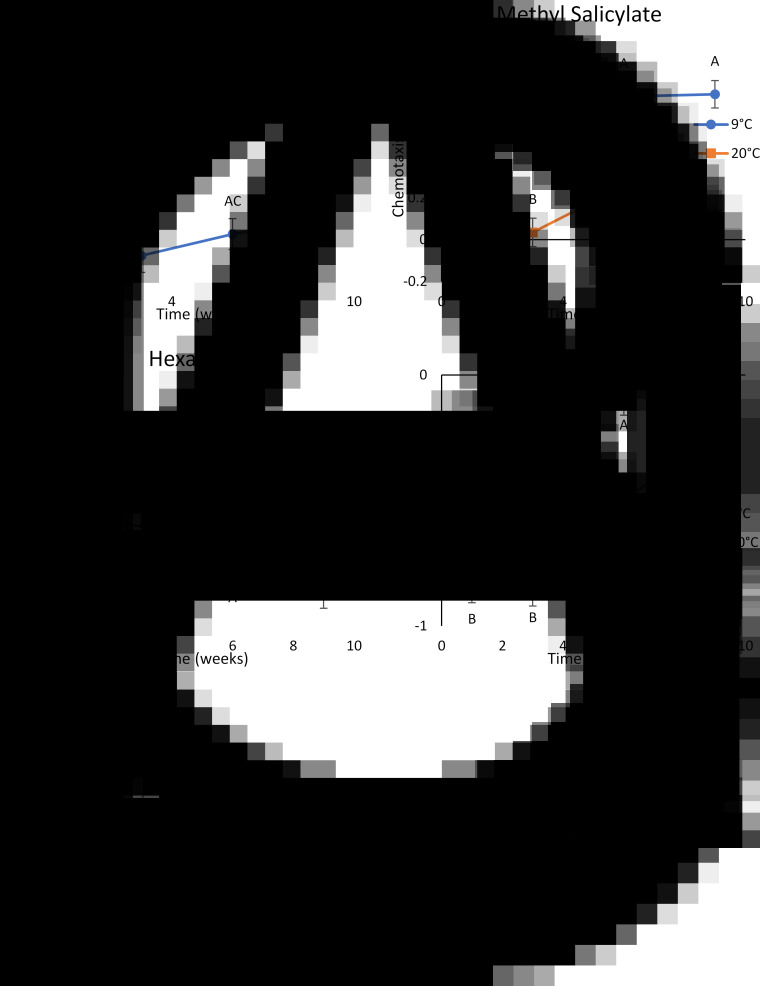
Chemotaxis index (mean ± s.e.) of *H. megidis* IJs after temperature conditioning at 9 and 20°C in response to 4 odorants. Within a panel, values accompanied by the same letter are not significantly different (*P* < 0.05, Dunn's multiple comparisons).

**Fig. 3.**
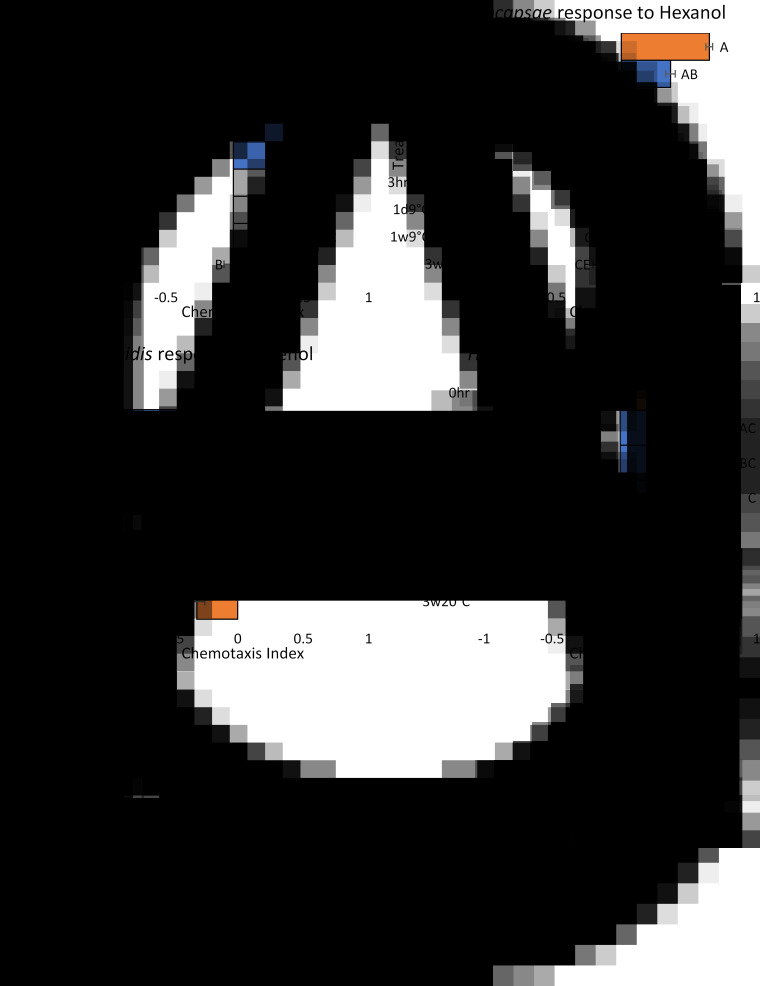
Chemotaxis index (mean ± s.e.) of *S. carpocapsae* (A, B) and *H. megidis* (C, D) IJs stored at 9°C for periods of up to 1 week and transferred into 20°C for the remainder of a 3-week period. Control IJs were kept at 9 or 20°C and tested at intervals stated. Within a panel, values accompanied by the same letter are not significantly different (*P* < 0.05, Dunn's multiple comparisons).

The results of chemotaxis experiments 1 and 2 are shown in Figs 1–3, respectively. *Steinernema carpocapsae* IJs were initially (time 0) strongly attracted to hexanol and methyl salicylate, strongly repulsed by prenol, and weakly attracted to acetone (Fig. 1). When stored at 9°C for 1 week, initially strong responses (CI > ±0.8) were completely reversed and remained so for the remainder of the 9°C storage period: IJs became repulsed by hexanol (Fig. 1C) and methyl salicylate (Fig. 1B) and highly attracted towards prenol (Fig. 1A). In contrast, the initially weak attraction (CI 0.3) towards acetone was intensified after 1 week at 9°C (Fig. 1D). The responses of IJs which remained at 20°C generally followed the same trend as those at 9°C but the change was more gradual: over time, IJs slowly became attracted to prenol (Fig. 1A), repulsed by methyl salicylate (Fig. 1B), and less attracted to hexanol (Fig. 1C). The response to acetone did not follow this trend; IJs were repulsed after 1 week at 20°C but the response returned to time 0 levels thereafter (Fig. 1D).

The reversal of chemotaxis observed following 1 week at 9°C seen in *S. carpocapsae* IJs in experiment 1 was explored over shorter time periods in experiment 2. Exposure to 9°C for only 3 h had a significant effect on the response to prenol, which changed from strong repulsion at time 0 to weak attraction after just 3 h at 9°C (Fig. 3A). A significant effect on the response to hexanol was first seen after 1 day at 9°C, and the response was reversed from positive to negative following 3 days at 9°C (Fig. 3B).

*Heterorhabditis megidis* IJs were initially (time 0) weakly attracted to methyl salicylate and hexanol and repulsed by prenol and acetone (Fig. 2). Storage at 9°C tended to enhance the response of IJs, which became more attracted to methyl salicylate (Fig. 2B) and more repulsed by prenol (Fig. 2A) and acetone (Fig. 2D). Exploration of shorter-term exposure to 9°C in experiment 2 showed that increased repulsion to prenol was seen already after just 1 day (Fig. 3C). Storage at 20°C tended to have the opposite effect to 9°C, and the greatest difference between storage temperatures was seen in the first 1–3 weeks (Fig. 2). The greatest difference between temperatures was for prenol, which also had the strongest time 0 response (CI −0.6; Fig. 2A), while little divergence was seen for hexanol which elicited a weak response (CI 0.1; Fig. 2C) at time 0.

### Chemotaxis of IJs stored for up to 1 week at 9°C and then transferred to 20°C

In experiment 1, the CI of *S. carpocapsae* IJs which were stored at 9°C for 1 week and then transferred to 20°C for the rest of the experiment tended to remain similar to the CI of IJs which remained at 9°C throughout (Fig. 1). The similarity persisted for the full 9 weeks in the case of prenol (Fig. 1A), but the responses began to diverge at 6 weeks in the case of methyl salicylate (Fig. 1C) and at 9 weeks for hexanol (Fig. 1B). The response to acetone of transferred IJs was intermediate between that of IJs stored exclusively at 9 or 20°C (Fig. 1D).

Cold-induced changes in chemotaxis towards prenol seen in *S. carpocapsae* by 3 h at 9°C were maintained and even intensified following transfer to 20°C (Fig. 3A). When tested after 3 weeks, the IJs which were transferred to 20°C after 3 h, 1 day or 1 week at 9°C had the same response as IJs left at 9°C for the full 3 weeks, differing significantly from the CI of IJs kept at 20°C for 3 weeks (Fig. 3A).

In contrast, although *H. megidis* IJs which were at 9°C for 1 day or 1 week had an altered (intensified) chemotatic response towards prenol, this was not maintained after subsequent storage at 20°C; the CI differed from that of IJs maintained for 3 weeks at 9°C but not from that of IJs that remained at 20°C throughout (Fig. 3C). A similar pattern was seen in response to methyl salicylate (Fig. 3D).

### Do all temperatures below culture temperature affect chemotaxis similarly?

**Fig. 4.**
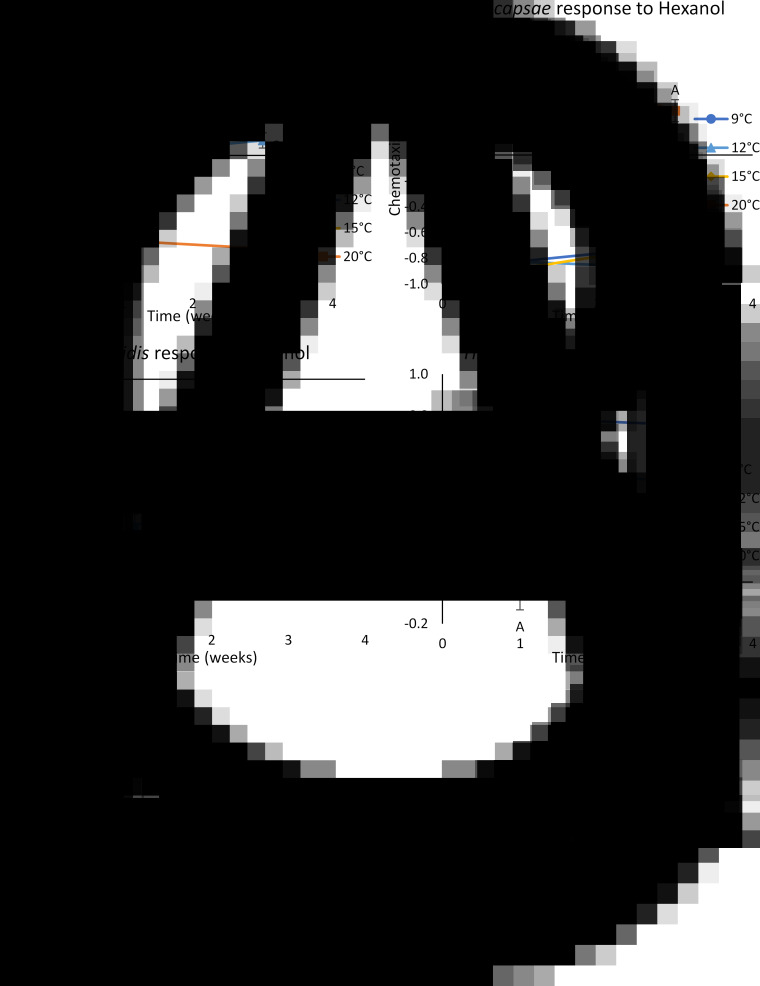
Chemotaxis index (mean ± s.e.) of *S. carpocapsae* IJs (A, B) and *H. megidis* IJs (C, D) and against a repellent (left) and an attractant (right), upon emergence from the host (time 0) and after storage at 9, 12, 15 and 20°C for 1 or 3 weeks. Within a panel, values accompanied by the same letter are not significantly different (*P* < 0.05, Dunn's multiple comparisons).

For *S. carpocapsae*, IJs stored at 9, 12 and 15°C all showed a similar CI, differing significantly (and with opposite valence) from that of IJs stored 20°C after 1 (hexanol) or 3 (prenol) weeks (Fig. 4A and B). Similarly, *H. megidis* IJs stored at 9, 12 or 15°C for 3 weeks had a similar CI for prenol that differed significantly from that of IJs stored at 20°C. However, chemotaxis towards methyl salicylate showed a more graded effect of storage temperature on *H. megidis*. After either 1 or 3 weeks, there was a significant difference between IJs stored at 9 and 20°C, with IJs stored at 12 and 15°C showing intermediate CIs (Fig. 4C and D).

### Effect of conditioning on freezing and desiccation tolerance of *S. carpocapsae*

**Fig. 5.**
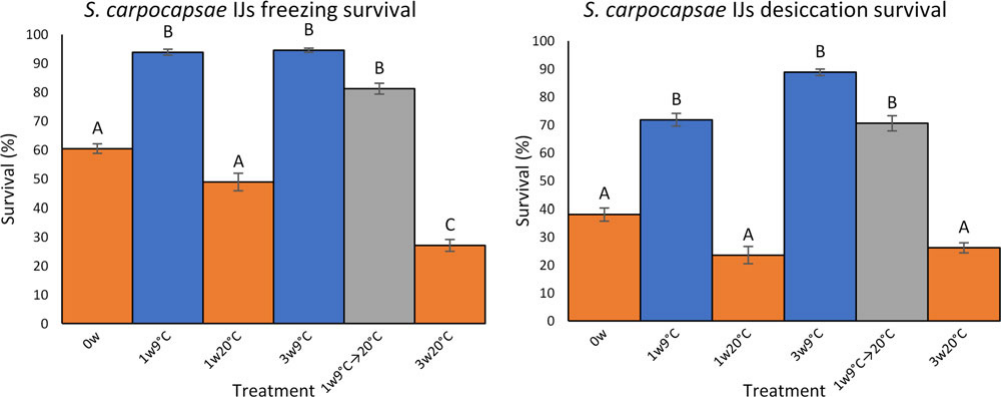
Survival (mean ± s.e.) of *S. carpocapsae* IJs exposed to freezing stress (−10°C for 6 h) or desiccation stress (75% RH for 5 days). IJs were either freshly emerged (time 0), stored at 9 or 20°C for 1 and 3 weeks, or stored at 9°C for 1 week and then swapped to 20°C for 2 weeks. Within a panel, values accompanied by the same letter are not significantly different (*P* < 0.05, Dunn's multiple comparisons).

*Steinernema carpocapsae* IJs stored at 9°C showed increased survival in both freezing (−10°C for 6 h) and in desiccation (75% RH for 5 days) assays relative to freshly emerged (time 0) IJs and those stored at 20°C, with significant differences in both cases (Fig. 5). IJs stored at 9°C for 1 week and transferred to 20°C for 2 weeks showed similarly high survival rates which were not statistically significantly different from those of IJs stored at 9°C for 1 or 3 weeks (Fig. 5).

### Response of *S. carpocapsae* proteome to storage conditions

**Fig. 6.**
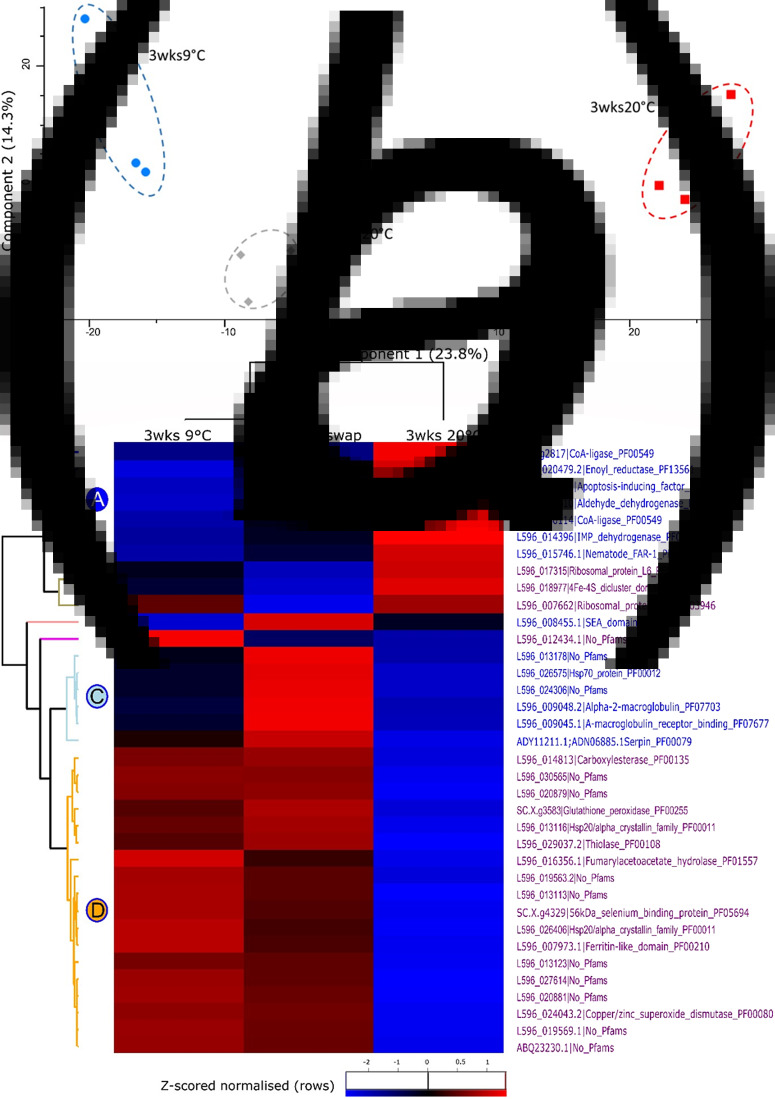
(A**)** PCA of proteins from *S. carpocapsae* IJs stored at 9°C for 3 weeks, at 9°C for 1 week and then swapped to 20°C for 2 weeks, and at 20°C for 3 weeks. (B) Heat map of *S. carpocapsae* All statistically significant proteins: 2-way unsupervised hierarchical clustering of the median *Z*-score normalized label-free quantification (LFQ) intensity values of all statistically significant proteins from IJs stored at 9°C for 3 weeks (left), at 9°C for 1 week and then swapped to 20°C for 2 weeks (middle), and at 20°C for 3 weeks (right). Differences in protein abundance are indicated by colour changes from low (blue) to high (red) protein abundance representative of changes in *Z*-score normalized log_2_-fold transformed LFQ intensity values.

In general, the proteome of *S. carpocapsae* IJs which were stored at 9°C for 1 week and transferred to 20°C for 2 weeks (temperature-swapped) was more similar to the proteome of IJs stored at 9°C than at 20°C for the full 3 weeks (Fig. 6).

In total, 2359 proteins were detected in the *S. carpocapsae* IJs, 36 of which were identified as statistically significant in a 1-way ANOVA. These 36 proteins (grouped by unsupervised hierarchical clustering in Perseus) are shown in a heat map (a visualization of the high and low abundance proteins present in the IJs; Fig. 6B) with further details shown in Table 1.

**Table 1.** Proteins identified in 2-way unsupervised hierarchical clustering of the median *Z*-score normalized label-free quantification (LFQ) intensity values of all statistically significant proteins (Benjamini–Hochberg false discovery rate 0.01), for *S. carpocapsae* IJs stored for 3 weeks, either at 9°C (3w9C) or 20°C (3w20C) throughout, or swapped from 9 to 20°C after 1 week (1w9C → 20C)

Relative abundance									
3w9C	1w9C → 20C	3w20C	Cluster	ID	BLAST annotation	Peptides	Mol. weight (kDa)	Intensity	MS/MS count
−0.72	−0.69	1.27	A	SC.X.g2817	Succinate-CoA ligase, alpha subunit	15	34.181	3.5 × 10^9^	229
−1.19	0.01	1.10	A	L596_020479.2	Hypothetical protein L596_020479	7	28.93	2.9 × 10^8^	49
−1.03	−0.15	1.14	A	L596_022932	Programmed cell death 8	10	70.283	2.5 × 10^8^	65
−1.07	−0.11	1.15	A	L596_013610	ALdehyde deHydrogenase	29	57.781	3.3 × 10^9^	297
−0.92	−0.11	1.26	A	L596_030114	Succinate--CoA ligase [GDP-forming] subunit beta, mitochondrial	7	46.012	3.0 × 10^8^	58
−0.84	−0.17	1.34	A	L596_014396	Inosine-5′-monophosphate dehydrogenase 1	7	34.475	3.5 × 10^8^	55
−0.89	−0.29	1.09	A	L596_015746.1	Fatty acid and retinol-binding protein	11	21.296	1.7 × 10^9^	90
−0.14	−1.01	1.06	B	L596_017315	60S ribosomal protein L9	11	21.254	2.9 × 10^9^	122
−0.27	−1.11	1.16	B	L596_018977	Putative NADH-quinone oxidoreductase subunit I	7	23.184	1.4 × 10^9^	59
0.49	−1.28	0.82	B	L596_007662	60S ribosomal protein L12	1	20.089	2.2 × 10^9^	63
−1.12	1.10	−0.17	X	L596_008455.1	SEA domain containing protein	5	84.611	3.4 × 10^8^	52
1.28	−0.52	−0.90	Y	L596_012434.1	Hypothetical protein L596_012434	3	51.288	2.3 × 10^8^	18
−0.13	1.23	−0.92	C	L596_013178	Hypothetical protein L596_013178	6	18.033	9.7 × 10^7^	21
−0.22	1.18	−1.08	C	L596_026575	Heat-shock protein	25	72.459	8.4 × 10^9^	342
−0.21	1.21	−1.08	C	L596_024306	Hypothetical protein L596_024306	8	33.817	6.1 × 10^8^	62
−0.32	1.24	−0.97	C	L596_009048.2	Alpha-2-macroglobulin family protein	30	86.756	2.3 × 10^9^	301
−0.25	1.25	−1.00	C	L596_009045.1	a-Macroglobulin complement component	21	99.788	7.2 × 10^8^	127
0.15	1.02	−1.24	C	ADY11211.1	Serine proteinase inhibitor	22	43.674	2.3 × 10^9^	203
0.63	0.78	−1.19	D	L596_014813	Carboxylesterase, type B domain-containing protein	34	77.629	8.4 × 10^9^	432
0.72	0.70	−1.29	D	L596_030565	Hypothetical protein L596_030565	5	14.902	7.4 × 10^8^	59
0.69	0.73	−1.35	D	L596_020879	LEA5 protein	3	7.4813	8.3 × 10^8^	28
0.44	0.89	−1.17	D	SC.X.g3583	Glutathione peroxidase	7	26.194	1.2 × 10^10^	119
0.52	0.84	−1.27	D	L596_013116	Hypothetical protein L596_013116	7	18.748	1.6 × 10^9^	62
0.44	0.81	−1.37	D	L596_029037.2	Acetyl-CoA C-acetyltransferase	11	42.561	5.8 × 10^8^	92
1.08	0.28	−1.27	D	L596_016356.1	CRE-FAH-1 protein	13	46.228	1.8 × 10^9^	118
0.85	0.46	−1.17	D	L596_019563.2	Hypothetical protein L596_019563	1	17.438	2.3 × 10^9^	102
0.86	0.46	−1.38	D	L596_013113	Hypothetical protein L596_013113	3	15.175	4.9 × 10^8^	22
0.87	0.42	−1.31	D	SC.X.g4329	Selenium-binding protein 1	15	50.706	8.8 × 10^8^	115
0.95	0.35	−1.36	D	L596_026406	CRE-HSP-12.1 protein	5	23.854	1.4 × 10^9^	73
0.96	0.39	−1.29	D	L596_007973.1	Ferritin-like protein	11	20.251	7.0 × 10^9^	130
0.61	0.49	−1.32	D	L596_013123	Hypothetical protein L596_013123	5	35.902	9.0 × 10^9^	195
0.80	0.50	−1.38	D	L596_027614	Hypothetical protein L596_027614	5	14.179	4.5 × 10^8^	44
0.82	0.54	−1.37	D	L596_020881	LEA5 protein	2	10.094	6.1 × 10^8^	20
0.73	0.59	−1.31	D	L596_024043.2	Extracellular superoxide dismutase [Cu-Zn]	5	18.899	2.4 × 10^9^	57
0.79	0.55	−1.31	D	L596_019569.1	Hypothetical protein L596_019569	42	68.331	7.1 × 10^9^	370
0.77	0.55	−1.28	D	ABQ23230.1	LEA1 protein	5	9.7546	5.2 × 10^9^	75

Cluster D consists of proteins which were detected at high abundance in the IJs stored at 9°C and in the temperature-swapped IJs, and low abundance in 20°C-stored IJs. This cluster is the largest of those detected, and consists primarily of molecular chaperones, such as heat-shock proteins (HSPs) and late embryogenesis abundant (LEA) proteins, and stress proteins such as glutathione peroxidase and copper superoxide dismutase involved in reactive oxygen species protection.

Clusters A and B are comprised of 10 proteins, which show the reverse pattern to cluster D: being detected in increased abundance at 20°C and decreased abundance in both the 9°C-stored and temperature-swapped IJs. Cluster A contains proteins related to fat binding and stress proteins while cluster B contains proteins related to the ribosome.

Cluster C consists of proteins which are highest in the temperature-swapped IJs, lowest in the 20°C-stored IJs and intermediate in the 9°C-stored IJs. This cluster contains a HSP, a serine protease inhibitor, macroglobulin-related proteins and various hypothetical proteins (Table 1). There were 2 ‘clusters’ which contained 1 protein each, without a clear function (Table 1).

### Highest and lowest abundance proteins

Statistically significant annotated proteins which were at least 5-fold increased or decreased in abundance (SSDAs) in the 9°C-stored and/or in the temperature-swapped IJs relative to the 20°C IJs are shown in Table 2. The proteins that showed the greatest increase in abundance in both the 9°C and temperature-swapped treatments were chaperone proteins, such as (*Caenorhabditis remanii* heat shock protein) CRE-HSP and LEA proteins which were 13.4- to 140.5-fold higher than in the IJs at 20°C for 3 weeks. Various enzymes were decreased in abundance relative to the 20°C-stored IJs including chitinase which was 25.6–39.2 times lower in the temperature-swapped and 9°C-stored IJs (Table 2). A number of proteins identified as SSDAs were not annotated (Supplementary Table 1), and these proteins may have important but unknown roles in temperature adaptation.

**Table 2.** Annotated statistically significant proteins 5-fold changed in abundance in *S. carpocapsae* IJs stored at 9°C for 3 weeks (3w9C) and those stored at 9°C for 1 week and transferred to 20°C (1w9C → 20C), relative to IJs stored at 20°C for 3 weeks

		Relative fold change *vs* 3w20C					
Protein ID	Blast annotation	3w9C	1w9C → 20C	Peptides	Mol. weight	Intensity	MS/MS count
L596_026406	CRE-HSP-12.1 protein	140.5	35.9	5	23.854	1.44 × 10^9^	73
L596_020879	LEA5 protein	93.6	95.8	3	7.4813	8.29 × 10^8^	28
L596_020881	LEA5 protein	61.2	33.5	2	10.094	6.11 × 10^8^	20
L596_015330	SaPosin-like protein family	60.0	43.9	4	10.214	1.16 × 10^9^	37
L596_019565.1	LEA2 protein	56.8	na	4	10.539	2.20 × 10^9^	45
L596_012217	PhosphoGlycolate phosphatase homolog	23.4	na	5	38.207	2.28 × 10^8^	18
L596_024791	Cystathionine beta-synthase	17.9	na	16	77.905	1.31 × 10^8^	43
ABQ23230.1	LEA1 protein	17.1	13.4	5	9.7546	5.25 × 10^9^	75
SC.X.g2587.2	Putative cystathionine gamma-lyase 2	7.4	8.7	5	42.673	1.07 × 10^8^	30
SC.X.g3323	Myosin regulatory light chain 1	7.3	na	4	18.941	8.40 × 10^7^	17
L596_019840	C. briggsae CBR-OSM-11 protein	7.2	na	3	28.814	2.74 × 10^8^	22
SC.X.g3305	C-1-tetrahydrofolate synthase, cytoplasmic	6.8	na	8	102.42	1.34 × 10^8^	48
L596_030616	Protein LSM12-like protein A	6.8	3.2	3	24.9	4.87 × 10^7^	17
L596_017887.4	ADP-ribose pyrophosphatase, mitochondrial precursor	5.7	5.7	4	17.112	1.22 × 10^8^	28
L596_024012	Thiamin pyrophosphokinase	na	45.7	9	30.405	2.58 × 10^8^	43
L596_028041	Serine/threonine-protein phosphatase PP1-alpha	na	7.0	2	19.921	1.13 × 10^8^	11
SC.X.g1861	Ani s 9 allergen precursor	na	5.3	2	14.755	9.04 × 10^7^	13
L596_026200	Ancylostoma secreted protein	na	−5.6	3	29.665	1.94 × 10^8^	26
SC.X.g6035	Ras protein let-60	na	−11.5	4	21.122	1.06 × 10^8^	15
L596_019533	Medium-chain specific acyl-CoA dehydrogenase, mitochondrial	−5.2	na	7	45.266	5.91 × 10^7^	27
L596_028677	Piwi domain protein	−6.0	na	5	100	5.03 × 10^7^	20
L596_017378	Trypsin-like serine protease	−6.8	−4.5	3	32.864	1.34 × 10^8^	22
L596_018471	Acetyl-coenzyme A synthetase 2, putative	−6.9	−4.0	4	78.513	7.81 × 10^7^	21
SC.X.g5447	Probable H/ACA ribonucleoprotein complex subunit 1-like protein	−8.2	−5.0	4	24.466	1.50 × 10^8^	23
SC.X.g3201	Short-chain dehydrogenase/reductase	−8.2	−3.6	2	29.235	7.42 × 10^7^	14
L596_023970	NAC domain containing protein	−11.8	na	4	21.879	1.57 × 10^8^	31
L596_015314	Chitinase class I	−39.2	−25.6	9	101.39	3.12 × 10^8^	33

Proteins which were identified but could not be annotated can be found in Supplementary Table 1.

## Discussion

Exposure to low temperatures had profound effects on the chemotaxis and stress tolerance of *S. carpocapsae* IJs, as expected based on previous research (Jagdale and Grewal, 2007; Lee *et al*., 2016). We also showed that these changes were accompanied by major proteomic reorganization and that the changes in chemotaxis, stress tolerance and proteome induced by low-temperature storage were largely maintained on return to culture temperature for 2 weeks. These findings have implications for how we understand the seasonal adaptations of these nematodes in their natural environment, but also how cold storage of mass-produced nematodes may impact their subsequent behaviour and survival following application for biocontrol purposes.

Storage at 9°C for 1 or 3 weeks enhanced *S. carpocapsae* IJs' tolerance to both freezing and desiccation. Cold acclimation is known to enhance the survival of nematodes, including *Steinernema* IJs, at sub-zero temperatures (Brown and Gaugler, 1996; Smith *et al*., 2008; Ali and Wharton, 2013), and there are also reports of cold acclimation enhancing desiccation tolerance (Jagdale and Grewal, 2007). Freezing and desiccation are closely linked environmental stressors for nematodes, both in the nature of the stress and the adaptations involved, with considerable cross-tolerance between the 2 stresses (Adhikari *et al*., 2010). The protective effect of cold acclimation has been attributed in part to the accumulation of trehalose and other low molecular weight polyols which play a protective role (Jagdale and Grewal, 2007; Ali and Wharton, 2015), while stress proteins are also important (Phadtare *et al*., 1999; Seybold *et al*., 2017). Recently, Wang *et al*. (2021) reported widespread transcriptional programming in eggs of the plant parasite *Meloidogyne incognita* acclimated at 4°C, including genes involved in lipid and carbohydrate metabolism, and HSPs.

In our study, the proteins which exhibited the greatest increase in abundance in cold-stored and temperature-swapped *S. carpocapsae* IJs relative to those maintained at the culture temperature were chaperone proteins. The highest abundance chaperone was CRE-HSP, a short heat shock protein (sHSP) found in *Caenorhabditis remanei*. Members of the sHSP family enhance cold and freezing stress tolerance (Sabehat *et al*., 1998; Pacheco *et al*., 2009; Wang *et al*., 2011). High levels of LEA proteins were detected in both 9°C-stored IJs and temperature-swapped IJs. LEA proteins, which have been identified in several nematode species (Solomon *et al*., 2000; Browne *et al*., 2004; Gal *et al*., 2004; Goyal *et al*., 2005), are disordered molecular chaperones which enhance an organism's survival under freezing stress (Solomon *et al*., 2000; Browne *et al*., 2002; NDong *et al*., 2002; Reyes *et al*., 2008; Anderson *et al*., 2015). LEA proteins interact with trehalose to facilitate the formation of bioglasses (Browne *et al*., 2004) which confer resistance to desiccation (Solomon *et al*., 2000) and freezing (NDong *et al*., 2002). The increased abundance of LEA proteins and HSPs induced by low-temperature storage may also have enhanced *S. carpocapsae* IJs' tolerance to desiccation (Close, 1996; Adhikari *et al*., 2009; Mizrahi *et al*., 2010; Hand *et al*., 2011).

The protein which was decreased to the greatest extent (at least 25-fold) in cold-stored relative to 20°C-stored IJs was chitinase, the enzyme responsible for degrading chitin. Chitinases have been detected in *Steinernema riobrave*-infected *G. mellonella* and are speculated to play a role in antifungal activity (Isaacson and Webster, 2002). Chitinases may also facilitate penetration of the insect cuticle and damage the host, expediting its death (Brandt *et al*., 1978; Osman *et al*., 2004; Hao *et al*., 2012). Thus, relatively low levels of chitinase in cold relative to warm-stored IJs may reflect the lower probability of IJs at low temperatures being infected by fungal pathogens, or of infecting an insect host, or both.

The ecological significance of increased stress tolerance and associated proteomic remodelling in response to cold is clear: a drop in temperature may indicate the onset of winter and the increased probability of unfavourable conditions including freezing. The changes in chemotaxis seen in response to cold are less easy to interpret, in particular the reversal of valence in which previously attractive substances become repulsive and vice versa. Similar phenomena were documented in detail by Lee *et al*. (2016), who showed that both culture and storage temperature affected the olfactory responses of *S. carpocapsae*, including a reversal of valence for several of the tested substances. In our experiments, the initial attraction of *S. carpocapsae* by hexanol and methyl salicylate, and repulsion by prenol, was completely reversed by 1 week at 9°C. Hexanol and methyl salicylate are released by plant roots (Roberts *et al*., 2019) and so attraction might help bring IJs to the rhizosphere and associated insects. Prenol was identified in *G. mellonella* infected with *Steinernema* spp. (Baiocchi *et al*., 2017), and was highly repulsive to several species of *Steinernema* and to *Heterorhabditis indica* (Baiocchi *et al*., 2017, 2019; Kin *et al*., 2019), and so the initial repulsion by prenol could help IJs avoid an already infected insect. When maintained at 20°C, the response of *S. carpocapsae* IJs to these 3 odours declined gradually, and reached reversal of valence after 6 weeks in the case of prenol and methyl salicylate. These age-related changes were exceeded by those of cold-stored IJs within a week. The attraction of *S. carpocapsae* IJs to prenol with age or after short-term exposure to cold may indicate the adoption of a more risk-prone strategy, with IJs prepared to enter an already infected insect. While infection of an already-occupied host lowers fitness due to competition (Koppenhöfer and Kaya, 1996; Ryder and Griffin, 2003; Blanco-Pérez *et al*., 2019), the increased protection from freezing and desiccation a cadaver confers (Lewis and Shapiro-Ilan, 2002; Perez *et al*., 2003) may offset those costs. Similarly, as an older IJ fails to find an uninfected host, invading an already occupied host may be preferable to continuing to wait for a fresh host. However, while prenol is an odorant given off by *Steinernema*-infected hosts, an infected host will likely release a complex variety of volatile and non-volatile chemicals, and an IJ's decision to infect will likely be the product of the blend rather than individual constituents.

Intriguingly, the changes in stress tolerance, protein expression and olfaction induced in *S. carpocapsae* by low temperature were not reversed following return to the culture temperature. IJs which were exposed to 9°C for 1 week retained their enhanced freezing and desiccation tolerance following storage at 20°C for 2 weeks, and the proteome of these temperature-shifted IJs was more similar to that of IJs maintained at 9°C than at 20°C. Similarly, a return of 9°C-stored IJs to 20°C did not revert their cold-induced attraction towards prenol back to the original repulsion. Even a short period at 9°C was effective in inducing long-term changes in chemotaxis, and these changes seem to have been completed during subsequent warm storage. Just 3 h at 9°C induced a significant change in response to prenol, from strong repulsion at time 0 to ambivalence when tested immediately, developing into strong attraction after a further 3 weeks at 20°C. Such rapid shifts in response valence are indicative of neuromodulatory changes rather than synaptic rewiring (Guillermin *et al*., 2017). In contrast to our findings with prenol, Lee *et al*. (2016) found that the alteration in olfactory response induced in *S. carpocapsae* IJs by a drop in temperature was reversed following return to the culture temperature. This may reflect differences in the temperatures used: 25 and 15°C in Lee *et al*. (2016) compared to 20 and 9°C in our experiments – as well as the specific odorants tested. In our assays, the maintenance of the cold-induced response following return to warm conditions was more clear-cut and distinct for prenol than for the other odours tested. Moreover, other factors such as age of the nematodes at the time of temperature shift can affect their olfactory plasticity (Lee *et al*., 2016).

Like *S. carpocapsae*, *H. megidis* IJs were initially repelled by prenol and attracted (though weakly) by hexanol and methyl salicylate. However, in contrast to *S. carpocapsae*, the response of *H. megidis* IJs to odours was generally accentuated rather than reversed by storage at 9°C, and cold-induced alterations were not retained by IJs after transfer back to 20°C. The 2 species also differed in the way that different storage temperatures affected their olfactory response: while for *S. carpocapsae*, all storage temperatures lower than the culture temperature of 20°C had a similar effect on olfaction, resulting in a reversal of valence, the effect of different storage temperatures on *H. megidis* IJs was more graded: IJs stored at 12 and 15°C exhibited an intermediate response between IJs stored at 20 or 9°C. As for *H. megidis* in our study, a shift to a lower temperature did not result in a change in the valence of *H*eterorhabditis *bacteriophora* IJs (Lee *et al*., 2016).

Our results support the conclusion of the comprehensive study by Lee *et al*. (2016) that plasticity in olfactory responses dependent on temperature and age is a broadly conserved trait across species of EPN, but that the exact nature of the changes varies between species and odorants. However, the ecological relevance of these changes remains to be understood. The behaviour of animals in response to single compounds in controlled laboratory assays may not be indicative of their response to blends of volatile and non-volatile chemicals encountered in the field (e.g. Webster *et al*., 2010). For IJs, the universally produced CO_2_ is the most important odour (Hallem *et al*., 2011; Dillman *et al*., 2012) and this remained attractive to most of the EPN species tested including *S. carpocapsae* and *H. bacteriophora*, irrespective of culture and storage conditions (Lee *et al*., 2016). Responses to live or infected insects and associated cues are the ultimate measure of how the prior storage temperature and time affect IJs in a way that is both ecologically relevant and of importance to biocontrol. The sophistication of the nematodes' responses to variables and stimuli applied singly under the highly artificial conditions of laboratory assays can only suggest the level of variation to be expected between individuals experiencing diverse combinations of factors in the soil environment.

While the ability of IJs to find and kill insects generally declines with age (Lewis *et al*., 1995*b*; Patel *et al*., 1997), there is also evidence of more complex effects of prior conditions. For *H. megidis*, conditions similar to those used in the present study have been shown to profoundly influence infectivity (a compound trait of movement towards and entry into insects), which increased with time, especially in IJs maintained at 9°C (Griffin, 1996; Fitters *et al*., 2001). Similar effects have been documented for steinernematids (Koppenhöfer *et al*., 2013; Guy *et al*., 2017; Yadav and Eleftherianos, 2018). Host-finding and infection in soil involve a series of steps, including dispersal, host-finding and host-recognition and acceptance (Lewis *et al*., 1995*a*; Griffin, 2015). Odour blends may increase the probability of IJs finding an insect but undirected dispersal and attraction to CO_2_ will result in many IJs arriving at it anyway, after which the decision is made as to whether to infect. A decreased responsiveness to host volatiles during cold storage of heterorhabditids and steinernematids can occur concomitantly with an increase in infectivity, which possibly indicates increased tendency to penetrate into a host following arrival (Dempsey and Griffin, 2002; Koppenhöfer *et al*., 2013).

While it is tempting to explain the cold-induced changes in chemotaxis as ecologically relevant, it is also possible that they are a by-product of altered thermotactic behaviour, such as migration to warmer regions of the soil, and/or tracking seasonal vertical movements of hosts within the soil (Villani and Wright, 1990). There is some overlap of neurons which mediate chemotaxis and thermotaxis, and these neurons may ‘memorize’ sensed temperatures (Clark *et al*., 2006; Kimata *et al*., 2012). While alterations in response to any specific odorant are difficult to interpret meaningfully in an ecological context, they are indicative of profound physiological changes brought about by temperature and by age as evidenced also in the proteomic data (present study; Lillis *et al*., 2022). Moreover, it is clear that the assays of chemo-attraction reflect the current status of the IJs, a product of their culture and storage conditions (present study; Lee *et al*., 2016) as well as learning (Willett *et al*., 2015). This extreme plasticity of olfactory responses needs to be taken into account in defining the role of specific volatiles for a species or strain of EPN.

Storage of *S. carpocapsae* and *H. megidis* IJs at temperatures lower than culture temperature affected each species in profoundly different ways. *Steinernema carpocapsae* IJs underwent reversals of valence in olfactory response while *H. megidis* IJs' chemotactic response to odours was generally intensified by exposure to low temperatures. Together with the proteomic profiling of Lillis *et al*. (2022), it appears that while both species are affected by storage time and temperature, *H. megidis* undergoes more graduated changes, in contrast to the dramatic changes demonstrated for *S. carpocapsae* when placed at 9°C. It is unclear to what extent the differences between the 2 species are a result of phylogeny or of species-specific adaptation to ecological factors. Similar studies on a larger number of species of each genus could help resolve this.

The proteome of *S. carpocapsae* IJs exhibited extensive remodelling upon exposure to 9°C, as previously shown by Lillis *et al*. (2022) with the novel finding that these changes were retained for weeks after their transfer to 20°C, as were changes in stress tolerance and chemotaxis. The most dramatic changes were found in chaperone proteins such as HSPs and LEA proteins which improve organisms' survival of freezing and desiccation. The demonstration that *S. carpocapsae* IJs' altered olfactory responses and enhanced stress tolerance induced by brief exposure to low temperature can be retained for weeks after their return to higher temperatures has implications both for laboratory testing and for their use as biocontrol agents. The rapid phenotypic adaptation of IJs also has implications for the interpretation of experiments testing genetic adaptation to different temperatures (Grewal *et al*., 1996). While we only explored the effects of storage conditions on the individuals that were exposed to those conditions, possible transgenerational (including epigenetic) effects of thermal history (Klosin *et al*., 2017; McCaw *et al*., 2020) should also be investigated. Moreover, the induction by brief cold exposure of profound changes that are maintained following the return of favourable conditions has resonances with certain forms of diapause and quiescence in parasitic nematodes and may share some common pathways with these poorly understood phenomena.

## Supplementary material

For supplementary material accompanying this paper visit https://doi.org/10.1017/S0031182022001445. click here to view supplementary material
