# Supplementary material for: Low-temperature exposure has immediate and lasting effects on the stress tolerance, chemotaxis and proteome of entomopathogenic nematodes
Source: Parasitology. 2022 Oct 14;150(1):15–28. doi: 10.1017/S0031182022001445 (PMC10090647; doi:10.1017/S0031182022001445)
Supplement: Supplementary file 1 [file S0031182022001445sup001.docx]

**Supplementary Table 1:** All statistically significant proteins fivefold changed in abundance in *Steinernema carpocapsae* IJs stored at 9°C for 3 weeks (3w9C) and those stored at 9°C for 1 week and transferred to 20°C (1w9C -> 20C), relative to IJs stored at 20°C for 3 weeks.

| **Protein ID** | **BLAST annotation** | **Fold change vs 3w20C** | | **Peptides** | **Mol. Weight** | **Intensity** | **MS/MS Count** |
| --- | --- | --- | --- | --- | --- | --- | --- |
|  |  | **3w9C** | **1w9C→20C** |  |  |  |  |
| L596_019569.1 | Hypothetical protein | 312.3 | 179.7 | 42 | 68.331 | 7.08E+09 | 370 |
| L596_026406 | CRE-HSP-12.1 protein | 140.5 | 35.9 | 5 | 23.854 | 1.44E+09 | 73 |
| L596_020879 | LEA5 protein | 93.6 | 95.8 | 3 | 7.4813 | 8.29E+08 | 28 |
| L596_020881 | LEA5 protein | 61.2 | 33.5 | 2 | 10.094 | 6.11E+08 | 20 |
| L596_015330 | SaPosin-like Protein family | 60.0 | 43.9 | 4 | 10.214 | 1.16E+09 | 37 |
| L596_019565.1 | LEA2 protein | 56.8 | na | 4 | 10.539 | 2.20E+09 | 45 |
| L596_030565 | Hypothetical protein | 36.2 | 32.7 | 5 | 14.902 | 7.41E+08 | 59 |
| L596_013113 | Hypothetical protein | 35.7 | 16.8 | 3 | 15.175 | 4.95E+08 | 22 |
| L596_012434.1 | Hypothetical protein | 29.9 | 2.2 | 3 | 51.288 | 2.26E+08 | 18 |
| L596_027614 | Hypothetical protein | 28.2 | 19.3 | 5 | 14.179 | 4.55E+08 | 44 |
| L596_012217 | PhosphoGlycolate Phosphatase Homolog | 23.4 | na | 5 | 38.207 | 2.28E+08 | 18 |
| L596_009145.1 | Hypothetical protein | 20.7 | 16.7 | 5 | 95.72 | 2.72E+08 | 89 |
| L596_024791 | Cystathionine beta-synthase | 17.9 | na | 16 | 77.905 | 1.31E+08 | 43 |
| ABQ23230.1 | LEA1 protein | 17.1 | 13.4 | 5 | 9.7546 | 5.25E+09 | 75 |
| L596_019563.2 | Hypothetical protein | 15.3 | 9.8 | 1 | 17.438 | 2.27E+09 | 102 |
| L596_027831.1 | Hypothetical protein | 10.1 | 7.8 | 8 | 51.877 | 6.22E+08 | 60 |
| SC.X.g2587.2 | Putative cystathionine gamma-lyase 2 | 7.4 | 8.7 | 5 | 42.673 | 1.07E+08 | 30 |
| SC.X.g3323 | Myosin regulatory light chain 1 | 7.3 | na | 4 | 18.941 | 8.40E+07 | 17 |
| L596_019840 | C. briggsae CBR-OSM-11 protein | 7.2 | na | 3 | 28.814 | 2.74E+08 | 22 |
| SC.X.g3305 | C-1-tetrahydrofolate synthase, cytoplasmic | 6.8 | na | 8 | 102.42 | 1.34E+08 | 48 |
| L596_030616 | Protein LSM12 -like protein A | 6.8 | 3.2 | 3 | 24.9 | 4.87E+07 | 17 |
| L596_013116 | Hypothetical protein | 6.5 | 9.5 | 7 | 18.748 | 1.62E+09 | 62 |
| L596_019570 | Hypothetical protein | 6.3 | na | 2 | 12.129 | 1.22E+08 | 12 |
| L596_017887.4 | ADP-ribose pyrophosphatase, mitochondrial precursor | 5.7 | 5.7 | 4 | 17.112 | 1.22E+08 | 28 |
| L596_024012 | Thiamin pyrophosphokinase | na | 45.7 | 9 | 30.405 | 2.58E+08 | 43 |
| L596_013178 | Hypothetical protein | na | 17.1 | 6 | 18.033 | 9.70E+07 | 21 |
| L596_028041 | Serine/threonine-protein phosphatase PP1-alpha | na | 7.0 | 2 | 19.921 | 1.13E+08 | 11 |
| SC.X.g1861 | Ani s 9 allergen precursor | na | 5.3 | 2 | 14.755 | 9.04E+07 | 13 |
| L596_017776 | Hypothetical protein | -5.0 | -13.0 | 2 | 14.45 | 1.19E+08 | 16 |
| L596_019533 | medium-chain specific acyl-CoA dehydrogenase, mitochondrial | -5.2 | na | 7 | 45.266 | 5.91E+07 | 27 |
| L596_012246 | Hypothetical protein | -5.5 | na | 4 | 13.671 | 8.92E+07 | 28 |
| L596_028677 | piwi domain protein | -6.0 | na | 5 | 100 | 5.03E+07 | 20 |
| L596_017378 | trypsin-like serine protease | -6.8 | -4.5 | 3 | 32.864 | 1.34E+08 | 22 |
| L596_018471 | acetyl-Coenzyme A synthetase 2, putative | -6.9 | -4.0 | 4 | 78.513 | 7.81E+07 | 21 |
| SC.X.g5447 | probable H/ACA ribonucleoprotein complex subunit 1-like protein | -8.2 | -5.0 | 4 | 24.466 | 1.50E+08 | 23 |
| SC.X.g3201 | Short-chain dehydrogenase/reductase | -8.2 | -3.6 | 2 | 29.235 | 7.42E+07 | 14 |
| L596_015745 | Hypothetical protein | -11.6 | -2.5 | 6 | 22.75 | 1.93E+08 | 26 |
| L596_023970 | NAC domain containing protein | -11.8 | na | 4 | 21.879 | 1.57E+08 | 31 |
| L596_020964 | Hypothetical protein | -13.4 | -11.0 | 5 | 19.028 | 3.80E+08 | 44 |
| L596_027332 | Hypothetical protein | -15.7 | na | 3 | 17.455 | 2.08E+08 | 22 |
| L596_015314 | Chitinase class I | -39.2 | -25.6 | 9 | 101.39 | 3.12E+08 | 33 |
